# The Immunomodulatory and Neuroprotective Effects of Mesenchymal Stem Cells (MSCs) in Experimental Autoimmune Encephalomyelitis (EAE): A Model of Multiple Sclerosis (MS)

**DOI:** 10.3390/ijms13079298

**Published:** 2012-07-24

**Authors:** Mohammed A. Al Jumah, Mohamed H. Abumaree

**Affiliations:** College of medicine, King Saud Bin Abdulaziz University for Health Sciences, King Abdullah International Medical Research Center, King Abdulaziz Medical City, National Guard Health Affairs, P.O. Box 22490, Riyadh 11426, Mail Code 1515, Saudi Arabia; E-Mail: jumahm@ngha.med.sa

**Keywords:** mesenchymal stem cells (MSC), experimental autoimmune encephalomyelitis (EAE), central nervous system (CNS), neurons, microglia, oligodendrocytes, neuroprotection

## Abstract

Mesenchymal stem cells (MSCs) are multipotent cells that differentiate into the mesenchymal lineages of adipocytes, osteocytes and chondrocytes. MSCs can also transdifferentiate and thereby cross lineage barriers, differentiating for example into neurons under certain experimental conditions. MSCs have anti-proliferative, anti-inflammatory and anti-apoptotic effects on neurons. Therefore, MSCs were tested in experimental autoimmune encephalomyelitis (EAE), an animal model of multiple sclerosis (MS), for their effectiveness in modulating the pathogenic process in EAE to develop effective therapies for MS. The data in the literature have shown that MSCs can inhibit the functions of autoreactive T cells in EAE and that this immunomodulation can be neuroprotective. In addition, MSCs can rescue neural cells via a mechanism that is mediated by soluble factors, which provide a suitable environment for neuron regeneration, remyelination and cerebral blood flow improvement. In this review, we discuss the effectiveness of MSCs in modulating the immunopathogenic process and in providing neuroprotection in EAE.

## 1. Introduction

Multiple sclerosis (MS) is a chronic, progressive inflammatory disorder of the central nervous system (CNS). It is characterized by myelin loss, various degrees of axonal pathology, and progressive neurological dysfunction [1]. The associated inflammatory plaque is the pathological hallmark of MS [2]. Experimental autoimmune encephalomyelitis (EAE) is the best-characterized animal model of MS [3]. The finding of inflammatory cells and their secreted molecules in the brain lesions of MS patients and of animals with EAE has supported the widely accepted notion that MS is mediated by pathogenic T cells, which react with myelin antigens, resulting in a larger degeneration of surrounding neurons [4]. These autoreactive T cells then migrate and cross the blood-brain barrier (BBB) to destroy the central neurons and their myelin sheaths as well as their axons.

The exact etiology of MS remains unknown. However, there are three key hypotheses that may explain the causes underlying MS, namely an immune response against the CNS, pathogen trigger and oligodendrocyte degeneration. In addition, genetic factors contribute to MS. Although the pathogenesis of MS is poorly understood, increasing evidence suggests that genetic and environmental factors may both contribute to the development of the disease [5]. Typically, MS affects young adults between 20 and 40 years of age [4,6]. MS shows a strong gender preference, with approximately 70 to 75% of all people with MS being female [4,6]. The incidence and prevalence of MS vary throughout the world, with at least one to two million individuals affected worldwide.

The key morphological characteristic of MS is the demyelination of nerve axons, which blocks or slows signal conduction at the site of demyelination [4,7]. MS patients suffer from a number of neurological symptoms, such as visual problems, changes in sensation, weakness, spasticity, acute/chronic pain, fatigue, depression and paralysis. Neurological symptoms develop when conduction blockade occurs concurrently in a considerable percentage of fibers within a particular neural pathway [7]. During clinical recovery, the inflammation and edema in the CNS resolve, and it is suggested that the restoration of CNS conductivity results from glial ensheathment and remyelination. In contrast, axonal loss is irreversible and may be the basis of neurological dysfunction in chronic MS.

Traditionally, there are four clinical forms of MS: relapsing-remitting MS (RRMS), secondary progressive MS (SPMS), primary progressive MS (PPMS), and progressive relapsing MS (PR). There is also another form of MS known as clinically isolated syndromes (CIS). These patients present with a single attack of the disease but are not yet diagnosed with MS. The most common form of MS is RRMS, which is associated with acute inflammatory episodes and a reduction in neurological functions [4]. Patients may experience some recovery between relapses, but 80% of RRMS patients progress to SPMS, which is associated with gradual loss of neurological functions and ascending paralysis [4].

In MS, remyelination and restoration of neuronal functions can be achieved by promoting endogenous mechanisms of neuronal repair or by transplanting exogenous myelinating cells [8]. However, long-term neuronal functional recovery requires regulation of the immunopathogenic process. The current treatments for MS are not completely effective because there is no effective therapy that can inhibit the functions of autoreactive T cells while inducing the remyelination and regeneration processes of neurons and thus prevent disability and irreversible axonal/neuronal damage [9,10]. In addition, there is no effective therapy for MS that can significantly modulate the functions of the cells of the CNS.

The pathogenic process of MS can be divided into inflammatory and degenerative phases. Therefore, to efficiently treat MS, it is necessary to develop a therapy that can specifically regulate the immune responses and that can also induce neuron regeneration. This will provide an effective regimen of immunomodulation and neuroprotection in MS patients. Several studies have shown many lines of evidence of neurodegeneration in MS, including the accumulation of amyloid precursor protein in neurons [11]; a reduction in the *N*-acetyl aspartate/creatine ratio, which reflects the degree of disability [12]; the finding of transected axons, which reflects the degree of inflammation within the active lesions [13]; damage to mitochondrial DNA and mitochondrial enzyme complexes [14]; and a reduction in axonal density in the white matter and spinal cords of MS patients [15,16].

Stem cell transplantation is a potential approach that can be used as a therapy to modulate the immunopathogenic process in MS to lead to neuron regeneration and treatment of the disease. Generally, stem cells can differentiate into various cell lineages with the ability to repair damaged tissue by reconstructing the tissue with new cells. The result is a recovery of lost functions, such as nerve conduction in patients with MS. Stem cells can perform their repair function by engrafting into the target tissue or by secreting paracrine factors that can trigger the repair pathways in the damaged tissue. Therefore, the utilization of stem cells in treating MS will rely on their ability to engraft into CNS tissues and then differentiate into neuron-like cells to replace the defective neurons or by secreting molecules that mediate the neuro-repair process in the CNS.

Generally, it is agreed that MSCs, which are derived from adult tissues, can attenuate the encephalitogenic manifestation of MS by suppressing the encephalitogenic T cells that mediate neuronal inflammation and damage [17–28]. The use of MSCs in EAE, the mouse model of MS, has shown that MSCs are able to modulate the immunopathogenesis of EAE and are also able to induce neuroprotection in EAE. Therefore, we will discuss the use of MSCs in EAE mice to investigate their effectiveness in attenuating the encephalitogenic process, possibly by inhibiting the functions of encephalitogenic T cell-mediated neuronal inflammation, neuronal demyelination and axon damage.

## 2. Experimental Autoimmune Encephalomyelitis (EAE)

The hypothesis that MS is an autoimmune disease was mainly based on the similarities observed between MS and its animal model, EAE. EAE is a demyelinating disease of the CNS that shares similar clinical and pathological features with MS. EAE is induced by immunizing animals with one of the myelin-derived antigens, such as proteolipid protein, myelin oligodendrocyte glycoprotein (MOG), or myelin basic protein (MBP) [29]. EAE is mediated by myelin-specific helper T cells, which are activated in the periphery and then translocate to the CNS, following permeabilization of the BBB [30,31]. Upon entering the CNS, T cells are reactivated by local and infiltrating active antigen presenting cells (APCs), such as dendritic cells, macrophages and microglia, resulting in inflammation and then subsequent neuronal demyelination and axonal damage [30,31].

Depending on the immunization protocol and the background of the mice, EAE can be induced in either an acute chronic progressive form or a relapsing-remitting form [32]. In addition, EAE can be induced by the adoptive transfer of activated myelin-specific helper T cells from EAE mice into naive recipient mice [33]. The EAE model is a useful tool with which to understand the immunopathogenesis of neuronal damage in EAE mice as well as to develop a therapy for MS [34,35].

### 2.1. Immune Cells in the Experimental Autoimmune Encephalomyelitis (EAE) Model

**Figure 1:**
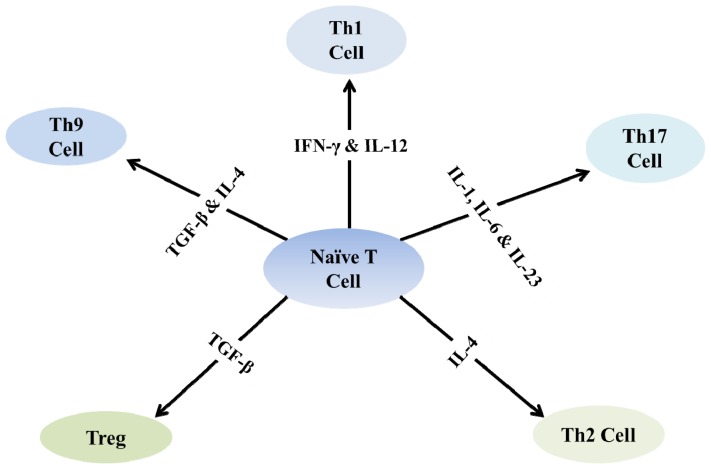
The differentiation of naïve T cells into different subtypes of T cells under the influence of cytokines. Interferon-γ (IFN-γ) and IL-12 convert naïve T cells into T helper 1 cells, whereas IL-4 promotes the development of T helper 2 cells. The differentiation of Th17 cells is promoted by IL-1, IL-6 and IL-23, and the differentiation of regulatory T cells requires TGF-β.

The inflammation present in MS and in its animal model, EAE, is largely mediated by autoreactive T cells that attack the CNS tissues. Dendritic cells that have been exposed to myelin-derived antigens secrete cytokines that induce the differentiation of naive T cells into effector T cells (Figure 1) in the lymph node. The differentiation of T helper 1 cells requires the presence of interferon-γ (IFN-γ) and IL-12, while IL-4 promotes the development of T helper 2 cells. In the mouse, the differentiation of Th17 cells is promoted by transforming growth factor-β (TGF-β), IL-6 and IL-21, whereas IL-1, IL-6 and IL-23 initiate Th17 differentiation in humans. The differentiation of regulatory T cells (Tregs) requires TGF-β [36].

However, it is still not clear how the inflammatory response is triggered in MS and EAE. It is possible that myelin antigens trigger the expansion of autoreactive lymphocytes in secondary lymphoid organs of susceptible MS patients and EAE mice. These immune cells then move from lymphoid organs into the circulation [37]. The expansion of inflammatory responses is also enhanced in MS patients and EAE mice due to poor Treg functioning [38]. Then, inflammatory cells adhere to and migrate across the BBB to infiltrate the tissues of the CNS, causing the characteristic inflammatory lesions surrounded by an area of neuronal demyelination and axonal loss [39]. There is evidence to suggest that the pathological heterogeneity in MS lesions is possibly a result of multiple distinct myelin-reactive effector T cells [40]. In addition, it has been noted that cytotoxic T-cells, which are present in MS lesions in significant numbers [41], may also contribute to tissue damage by attacking oligodendrocytes and by transecting axons [42,43].

#### 2.1.1. T Helper 1 (Th1) and T Helper (Th17) Cells

The inflammatory role of Th1 cells in MS and EAE has been established. It has been reported that Th1 cells secrete inflammatory cytokines in the peripheral blood and in the CNS of affected subjects [44,45]. In addition, microglia activate Th1 cells in the CNS, which in turn activate macrophages to mediate myelin damage through the release of toxic mediators, such as tumor necrosis factor-alpha (TNF-α) [46]. Moreover, the adoptive transfer of myelin-specific CD4^+^ Th1 cells into naïve recipient mice can induce EAE in these mice [47–54]. Therefore, MS research was primarily based on IFN-γ-producing T cells because myelin-specific CD4^+^ Th1 cells were sufficient to induce EAE in mice. Several studies have demonstrated that altering IFN-γ production in the myelin-specific T cells prior to their transfer into recipient mice could decrease the encephalitogenic capacity of these CD4^+^ Th1 cells [52,55,56].

The essential role of IFN-γ in autoimmune encephalomyelitis was further assessed. It was shown that IFNγ-deficient mice were susceptible to EAE, and the disease appeared to be more severe in these mice as compared to control mice [57–59]. This result was further confirmed by using antibodies to neutralize IFN-γ [58,60]. In addition, the number of myelin-specific CD4^+^ T cells was shown to be increased in IFN-γ-deficient mice [61]. These results suggest that CD4^+^ Th1 cells can also induce EAE via a mechanism that is independent of IFN-γ. Thus, other cytokines may influence the pathogenic capacity of T cells.

Therefore, the focus of EAE research was shifted to investigations of the role of IL-12 in EAE because it is essential for the differentiation of Th1 cells. The p40 and p35 proteins, which together comprise IL-12, were deleted in mice, and EAE was then evaluated. It was shown that IL-12p40-deficient mice failed to develop EAE; however, EAE was induced in IL-12p35-deficient mice [62,63]. Because IL-12p40 is also a component of IL-23, the role of IL-23 was also evaluated in EAE mice. Because IL-23 also includes the p19 protein, the role of this protein in EAE pathogenesis was also evaluated in EAE mice. Interestingly, IL-12p40- and IL-12p19-deficient mice were resistant to EAE [64]. Accordingly, this result demonstrates that IL-23 and not IL-12 is crucial for the induction of EAE. In addition, IL-23 was found to promote the expansion of myelin-specific IL-17+ T cells, and these IL-17+ T cells were found to induce EAE [65]. Moreover, these Th17 cells produce IL-17, IL-21, IL-9, IL-22 and TNF-α and promote inflammation in EAE [53,65–67]. These findings led to the speculation that myelin-specific Th17 cells were the primary encephalitogenic T cell population in EAE and, possibly, in MS.

Several studies further confirmed the substantial role of Th17 cells in mediating neuronal immunopathogenesis in MS patients and in EAE mice. It has been reported that the number of Th17 cells increases in both the peripheral blood and the brain of MS patients [68,69]. In addition, it was shown that the brain endothelium of MS patients expresses high levels of the IL-17 receptor, and its ligand, IL-17, increases the permeability of the BBB to inflammatory cells [70]. Moreover, recent studies on EAE suggest that the initial inflammatory event in the CNS involves the migration of Th17 cells from the peripheral blood to the spinal fluid [71]. Subsequently, Th17 cells activate the BBB and allow the entry of Th1 cells into the CNS. The blockade of IL-17 was shown to reduce disease severity in EAE [72,73]. In addition, it was demonstrated that the disease severity of EAE was markedly reduced in IL-17-defective mice [74,75]. Moreover, it was reported that Th17 may induce EAE in mice via IL-9 because it was found that the neutralization of IL-9 can attenuate EAE [76]. Furthermore, the Th17 cells that induce EAE in mice were also found to be dependent on IL-1 because IL-1R-defective mice exhibited impaired Th17 cell activity and were also resistant to EAE induction [77].

Collectively, these studies demonstrated the significant roles of both Th1 and Th17 responses in mediating the immunopathogenesis of MS and its animal model, EAE. Therefore, Th1 and Th17 cells could be potential targets for the development of therapies for MS that modulate the immunopathogenic process to induce neuron regeneration.

#### 2.1.2. T Helper 9 (Th9) Cells

TGF-β and IL-4 induce the development of T helper 9 cells, which produce IL-10 and IL-9 [78,79]. Although Th9 cells produce IL-10, they do not perform immunosuppressive functions. Th9 cells are different from Th1, Th17, and Foxp3-inducible Treg cells [78]. Recently, it was reported that IL-9 contributes to the induction of allergy and asthma [80]. In addition, it was shown that T helper 9 cells can also induce colitis and peripheral neuritis [6]. Moreover, MOG-specific Th9 cells were also shown to induce EAE and peripheral neuritis. Furthermore, IL-9, which is produced by the MOG-specific Th9 cells, can also activate mast cells, which induce demyelination [41].

#### 2.1.3. γδ T Cells

γδ T cells provide the first line of defense against infection at mucosal sites. γδ T cells directly recognize ligands induced by stress, inflammation or infection. γδ T cells are also involved in both innate and adaptive immunity, and they play a role in both MS and EAE. The detection of γδ T cells in acute MS brain lesions has confirmed their potential role in the neuroimmunopathogenic process in MS [81]. This was further confirmed by the finding of these cells in the cerebrospinal fluid of MS patients [82]. Recently, IL-17 was shown to be produced by γδ T cells during infection [83,84]. In addition, these IL-17-producing γδ T cells were also reported to be present at high frequency in the brains of mice with EAE, and these cells increased the susceptibility of mice to EAE [85]. Thus, these studies confirmed the pathogenic role of γδ T cells in EAE. However, these cells exhibited both protective and pathogenic roles in the EAE model [86–93]. These conflicting results were attributed to the use of different mouse strains and the methods used to deplete γδ T cells.

#### 2.1.4. Regulatory T Cells (Tregs)

Regulatory T cells (Tregs) include natural and inducible Treg cells. Natural Tregs (nTregs) are CD4^+^ CD25^+^ T cells, which develop in the thymus and then migrate to the periphery to perform their key role in immune homeostasis [94–96]. Adaptive Tregs, including Tr1, Th3 and various subsets of CD8^+^ Tregs (as discussed below), are derived in the periphery from naive T cells stimulated by antigen under the influence of the immunosuppressive cytokines IL-10 and TGF-β [97].

Various types of Treg cells were shown to play crucial roles in the regulation of autoimmune inflammation in MS patients and in EAE mice. It was reported that Tr1 responses and the frequency of nTreg cells are reduced in MS patients [98–101]. In mice, Tregs can control the development and severity of EAE. In addition, it was reported that transgenic mice expressing a T cell receptor specific for myelin antigen develop EAE, whereas non-transgenic CD4^+^ T cells prevented EAE, suggesting a suppressive role for CD4^+^ Treg cells [102,103]. Moreover, the transfer of CD4^+^CD25^+^ T cells into EAE mice can reduce the severity of the disease [104].

#### 2.1.5. CD8^+^ T Cells

Several studies demonstrated that CD8^+^ T cells have a crucial role in the pathogenesis of MS and EAE. CD8^+^ T cells were found in significant numbers in MS patients, as well as in EAE mice [41,105,106]. In addition, these cells contribute to tissue damage by attacking oligodendrocytes and by transecting axons [42,43]. However, a regulatory role for CD8^+^ T cells was also demonstrated in EAE mice. It was demonstrated that EAE is more severe in mice deficient in CD8^+^ T cells [106], and distinct subpopulations of CD8^+^ Treg cells, including CD8^+^ CD28^−^ and CD8^+^ CD122^+^ Treg cells, which can regulate EAE, were also identified [107,108].

Recently, CD8^+^CD28^−^ regulatory T cells have been characterized in humans. It has been shown for the first time that there is a population of CD8^+^CD28^−^ suppressor T cells that can suppress the alloreactivity of T helper cells [109–113]. The role of CD8^+^CD28^−^ regulatory T cells in a chronic model of EAE was also investigated, and it was shown that CD8^+^ T cells lacking CD28 expression are responsible for the regulatory functions of CD8^+^ T cells in EAE mice. These regulatory T cells can inhibit the activation of APCs and thus inhibit the activation of the encephalitogenic CD4^+^ Th1 cells [107].

The role of CD8^+^CD122^+^ regulatory T cells in EAE was also established. CD8^+^CD122^+^ regulatory T cells, which produce IL-10, can directly control CD8^+^ cells by suppressing CD8^+^ T cell production of IFN-γ, as well as by inhibiting the proliferation of CD8^+^ T cells [114,115]. It was demonstrated that the depletion of CD122^+^ cells can increase the duration of EAE symptoms in affected mice, while the transfer of CD8^+^CD122^+^ regulatory T cells into EAE mice can dramatically diminish the symptoms in EAE mice, thus demonstrating the protective role of CD8^+^CD122^+^ T cells in EAE [108]. In addition, in β2 microglobulin^−/−^ mice, where CD8^+^ T cells do not develop due to MHC I deficiency, a regulatory role for CD8^+^ T cells was demonstrated in EAE [116]. Moreover, CD8^+^ T cells isolated from EAE-recovered mice specifically inhibit MBP-activated CD4^+^ T-cells *in vitro*, and their depletion was also followed by recurrence of EAE. The suppressive function of these CD8^+^ T cells is restricted by the MHC I-like Qa-1 molecule (murine homologue of the human HLA-E), and the adoptive transfer of these cells prevented disease in MBP-immunized mice [117–119]. The failure of resistance to EAE in Qa-1-deficient mice is associated with the escape of Qa-1-deficient CD4^+^ cells from CD8^+^ T-cell suppression [120]. The suppressive role of CD8 T cells was further confirmed. It was shown that CD8^+^ Tregs, which express latency-associated peptide (LAP), can suppress myelin oligodendrocyte glycoprotein-specific immune responses in EAE via a mechanism that requires both IFN-γ and TGF-β [121]. These results provide evidence that CD8^+^ T cells are important in both inducing resistance to EAE and in abrogating recurrent relapsing episodes of pathogenic autoimmunity *in vivo*.

These studies demonstrated the essential roles that T cells may play in the immunopathogenesis of MS and its animal model, EAE. Therefore, T cells, including Th1, Th17, Th9, γδ T, CD4 regulatory T cells, CD8 T cells and CD8 regulatory T cells, are potential targets for the development of therapeutic strategies that aim to control T cell mediation of autoimmune processes in the EAE mouse model. Such therapeutic can be further developed into effective treatments for MS. However, recent studies revealed that MS is not only a T cell disease, as B cells were shown to significantly contribute to the disease. An increasing number of reports have demonstrated that B lymphocytes have an important role in the pathogenesis of MS. This includes the presence of B cells, plasma cells, autoantibodies and complement deposition in the blood, CSF and in CNS lesions in the majority of MS patients [122,123]. Most importantly, these autoantibodies contributed to the demyelination process [124–127], and they were reactive against myelin proteins and neurons [128,129]. In addition, a complement-mediated lysis of B cells effectively reduced MS disease activity, thus further confirming the pathogenic role of B cells in MS [130].

In addition, B cells function as APCs by presenting antigen to helper T cells. It was shown that B cells act as APCs in the periphery in MS patients [131]. Therefore, B cells may support the response of T helper cells in the periphery by activating naïve autoreactive cells and subsequently allowing them to enter the CNS. Thus, B cells may also have a regulatory function through direct contact with T cells. The costimulatory pathway, including CD154-CD40 and CD28-B7, is essential to inhibit the proliferation of T cells through the release of cytokines, such as IL-10 and TGF-β. It was reported that IL-10-producing B cells are deficient in MS patients [132]. Therefore, B cells can be one of the potential targets in MS research for the development of an effective therapy. Simply stated, we can inhibit the functions of naïve T cells via a mechanism that is dependent on the control of B cell functions.

Dendritic cells, which are a group of professional APCs, modulate adaptive immune responses [133]. Generally, inflammatory conditions induce the maturation of dendritic cells, which then induce T cell polarization [134]. Human immature dendritic cells can induce IL-10-producing Tregs and can also induce T-cell anergy [135]. Additionally, regulatory dendritic cells, which have a phenotype different from that of immature DCs, can promote peripheral tolerance and regulatory T cell development [135]. In MS, dendritic cells are among the cells that can infiltrate the CNS [136]. In addition, in SPMS, patients have high numbers of circulating mature dendritic cells compared with RRMS patients [137]. Therefore, dendritic cells are another potential therapeutic target for MS research with regard to the regulation of their functions, which will result in the modulation of the functions of autoreactive T cells, thereby improving or curing the disease.

Macrophages are also important in MS pathogenesis. Perivascular CNS macrophages can be activated by T helper 1 cytokines in MS lesions [138]. Macrophage activation can be either proinflammatory or anti-inflammatory, depending on the cytokine exposure. Macrophages exposed to IFN-γ are classified as proinflammatory M1 macrophages and are likely to contribute to myelin damage by phagocytosis and the release of neurotoxic mediators. Myelin- and neuroantigen-containing APCs were described in the cervical lymph nodes of healthy individuals, and these CNS antigen-containing APCs are increased in MS patients. These data suggest that myelin and neuronal antigens released from damaged CNS tissue may be captured by the CNS APCs and migrate into the cervical lymph nodes. Interestingly, neuronal antigens are presented by proinflammatory APCs, whereas myelin antigens are presented by anti-inflammatory APCs. Thus, pathogenic and regulatory CNS-specific T cells may be differentiated in the cervical lymph nodes [139]. These data provide another therapeutic pathway that can be pursued in the development of MS treatment. The modulation of macrophage functions would aid in regulating the autoimmune response of T cells, which would offer direct or indirect suppressive mechanisms by which to control the autoimmune responses and neuronal damage in MS patients.

Clearly, B cells, dendritic cells and macrophages can all contribute to the pathogenesis of MS; therefore, they are potential targets that should be pursued to develop effective therapies for MS. However, there is limited information regarding the modulating effects of MSCs on the functions of B cells, dendritic cells and macrophages in EAE. Therefore, our focus in this review will be on the effectiveness of MSCs in attenuating the encephalitogenic manifestation of EAE by suppressing the functions of the autoreactive T cells that mediate neuronal inflammation and damage.

## 3. Mesenchymal Stem Cells (MSCs)

Stem cells are specialized cells that are capable of self-renewal and multilineage differentiation. They fall into the two broad categories of embryonic stem cells (ESCs) and adult stem cells (ASCs). ESCs are pluripotent and differentiate into cell derivatives of the three germ layers: endoderm, ectoderm and mesoderm. ESCs are derived from the inner cell mass of the blastocyst; therefore, their use in cell-based therapy is controversial because of blastocyst destruction [140–143]. In contrast, the use of ASCs in cell-based therapy is less controversial because they are obtainable from a wide range of tissues, such as bone marrow, adipose tissue, placenta and umbilical cord. One important subset of ASCs is MSCs, which are multipotent cells that differentiate into mesenchymal cell lineages, including adipocytes, osteocytes, chondrocytes and myocytes [144,145]. However, MSCs can “transdifferentiate” and thereby cross lineage barriers, differentiating into different cell types, such as neurons (see below).

Mesenchymal stem cells have immunosuppressive properties that make them a therapeutic option that can be used to modulate the immunopathogenesis of multiple sclerosis and its animal model, EAE. Therefore, the effectiveness of MSCs in attenuating the encephalitogenic manifestation of EAE by suppressing the functions of the autoreactive T cells that mediate neuronal inflammation and damage has been examined.

### Immunosuppressive Characteristics of Mesenchymal Stem Cells

**Figure 2:**
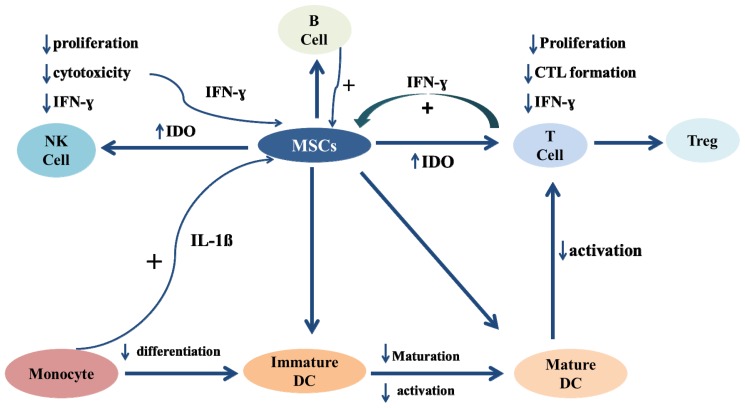
Immunomodulatory effects of MSCs on immune cells, including T cells, NK cells, B cells, monocytes and dendritic cells (DCs). MSCs can inhibit the proliferation and the cytotoxic functions of T and NK cells. MSCs can also modulate the functions of B cells. In addition, the differentiation of monocytes into immature DCs is inhibited by MSCs. Moreover, the maturation of DCs and their ability to activate T cells are also affected by MSCs.

Several studies have confirmed the immunosuppressive characteristics of MSCs. It was found that MSCs express MHC-I but lack the expression of MHC-II and costimulatory molecules [146–161]. In addition, MSCs suppress the immune responses of allogeneic lymphocytes [162,163]. In a mixed lymphocyte reaction, baboon or human bone marrow derived MSCs (BMMSCs) can inhibit the proliferation of allogeneic lymphocytes (Figure 2) [162,163]. In addition, human BMMSCs can also suppress the proliferative functions of T cells stimulated by antibodies to CD3 or CD28 [164]. Moreover, murine and human BMMSCs can inhibit the proliferation of lymphocytes stimulated with anti-CD3, IL2, IL7 or IL15 *in vitro* [165,166]. This inhibitory effect was shown to be partially mediated by IFN-γ [166]. Similarly, human placental MSCs and amniotic membrane MSCs can suppress the proliferation of allogeneic lymphocytes [146,148,149,167–170]. Furthermore, fetal liver MSCs can inhibit mitogen-stimulated lymphocytes [171,172], and it was similarly demonstrated that adipose-derived MSCs can inhibit T cell proliferation [18]. Moreover, it was shown that dental pulp MSCs (DP-MSCs) can suppress the proliferation of peripheral blood mononuclear cells [161]. Collectively, these studies showed that MSCs derived from different sources are immunosuppressive through the inhibition of the proliferation of allogeneic lymphocytes.

In addition, it was shown that MSCs can modulate the functions of both T and B lymphocytes. MSCs can inhibit the production of TNF-α and IFN-γ by CD4^+^ T and CD8^+^ T cells, whereas they can upregulate the expression of IL-10 and restore the secretion of IL-4 by CD4^+^ and CD8^+^ T cells [173] (Figure 2). In addition, fetal liver MSCs can down-regulate the production of IFN-γ and can increase the secretion of IL-10 in stimulated T cells [172]. Similarly, it was reported that MSCs derived from adipose tissues can enhance the secretion of IL-4, IL-5, and IL-10 by T cells [18]. In contact cultures, human MSCs were shown to suppress the *ex vivo* expansion of γδ T cells without modulating their cytotoxic function [174].

In addition, BMMSCs were found to selectively suppress the proliferative activities of both T and B lymphocytes via a mechanism that is mediated by programmed death 1 inhibitory molecule (PD-1) and its ligands PD ligand-1 (PD-L1) and PD ligand-2 (PD-L2) [175,176]. Moreover, BMMSCs can suppress the immune function of B cells stimulated by anti-CD40 or IL-4 [177]. This inhibitory effect of BMMSCs on B cells was also confirmed in other studies. It was shown that human BMMSCs can suppress the proliferation, differentiation and chemotactic activities of B cells [178,179]. Similarly, human placental MSCs can also suppress the immune responses of different populations of immune cells, including CD4^+^ and CD8^+^ T cells [148].

CD8^+^ cytotoxic T lymphocytes (CTLs) and natural killer (NK) cells are effector cells with cytotoxic activities that can eliminate cancer or infected cells. CTLs are stimulated following their interaction with antigenic peptides expressed on MHC class I molecules. Human BMMSCs are recognized as targets by pre-stimulated alloreactive CTLs, and they can suppress the differentiation of CTL precursors into CTL effectors through the secretion of suppressive factors [180,181].

NK cells that are constitutively cytotoxic against allogeneic cells cannot lyse MSCs [148,181]. However, NK cells that are stimulated with IL-2 can lyse MSCs [182,183]. In addition, NK cells stimulated with IL-2 and IL-15 can lyse MSCs [184]. Therefore, these data on the ability of NK cells to lyse MSCs are contradictory. In addition, a recent study showed that CD8^+^ T and NK cells can lyse allogeneic MSCs [185]. Therefore, more research is necessary to study the susceptibility of MSCs to lysis by immune cells because this knowledge is indispensable for the development of an effective and safe MSC therapy. However, it is possible that MSCs have a transient effect on the inflammatory milieu in graft *versus* host disease (GVHD) because it was shown that MSCs can have long-lasting effects by passing on some of their effects to other cell types, such as regulatory T-cells [186,187]. Thus, this result indicates that the long-term effectiveness of MSCs would not be diminished if MSCs are lysed soon after infusion.

In addition, human BMMSCs can inhibit the proliferation of NK cells and the cytolytic activity of NK cells [188]. Moreover, Human BMMSCs can inhibit the production of IFN-γ by NK cells [188]. However, another study showed that human BMMSCs can significantly increase the secretion of IFN-γ by NK cells [183]. This inconsistency was attributed to the different effects that MSCs could have on NK cells. This possibility may depend on whether NK cells are triggered by IL-2 [183]. Another possibility is that the ratios of NK cells to MSCs used in different experimental settings may have differential activating or inhibitory effects on NK cells by MSCs. It is difficult to determine *in vivo* whether one or many NK cells interact simultaneously with an individual stem cell or *vice versa*.

Regarding the mechanism underlying the immunosuppressive function of MSCs, several reports suggest that cell-cell contact is not a compulsory requirement for the suppression of immune cell functions by MSCs [189–191]. Therefore, MSCs must produce soluble factors that mediate their immunosuppressive functions on immune cells. Several soluble factors were detected in the culture medium of MSCs, including stem cell factor (SCF), IL-6, IL-8, IL-10, IL-12, IFN-γ, PGE_2_ (prostaglandin E_2_), vascular endothelial growth factor (VEGF), macrophage colony-stimulating factor (M-CSF), hepatocyte growth factor (HGF) and transforming growth factor -β1 (TGF-β1) [146,161,188,192,193].

The immunosuppressive capacity of MSCs *in vivo* was also confirmed in various studies [191]. It was reported that the intravenous injection of MSCs can prolong the survival of an allogeneic skin graft in baboons [162]. Likewise, the injection of murine BMMSCs into mice can stimulate the survival of allogeneic skin grafts in mice [194]. In addition, it was shown that MSCs were not rejected following their transplantation into allogeneic immunocompetent mice [190]. However, the subcutaneous injection of melanoma cells resulted in tumor growth in allogeneic recipients only when MSCs were co-injected [190]. Although the possible side effects of immunosuppression induced by MSCs need to be investigated in more detail, the effectiveness of MSCs for many therapeutic applications remains of great interest. Recently, it was revealed that the injection of mouse MSCs prolonged the survival of skin transplants in mice [195]. In addition, the immunosuppressive effect of human MSCs on the severity of bleomycin-induced inflammation and fibrosis in an animal model was evaluated. The presence of transplanted MSCs reduced the neutrophil infiltration and significantly decreased the inflammation, as well as the severity of lung fibrosis, in mice treated with allogeneic or xenogeneic placenta-derived cells [196].

The therapeutic efficacy of MSCs in the murine model of MS, EAE, was also reported (see below). In addition, the intravenous injection of baboons with autologous or allogeneic baboon MSCs together with hematopoietic progenitor cells facilitated a faster hematopoietic recovery [197]. This result was further confirmed by a recent study showing that allogeneic BMMSCs can reduce the severity of GVHD in an F1 model of acute GVHD [198].

Therefore, the immunosuppressive features of MSCs, together with their ability to differentiate into neuronal lineages, support the use of MSCs in EAE to modulate the immunopathogenic process underlying the neuronal damage, as well as to offer neuroprotection in EAE, to develop therapeutic strategies for MS.

## 4. The Use of MSCs in Clinical Experimental Autoimmune Encephalomyelitis (EAE)

### 4.1. Immune Modulatory Effect of MSCs in EAE

Several studies showed that MSCs exert immunoinhibitory functions on immune cells *in vitro* and *in vivo* [191]. These characteristics, together with the ability of MSCs to differentiate into neuron-like cells [199] and migrate to the CNS [200], promoted the use of MSCs in EAE treatment. Several studies confirmed the differentiation of MSCs into neurons. Rat and human BMMSCs were shown to differentiate into neurons *in vitro* [201,202]. The differentiated cells expressed a variety of neuron-specific markers, including neuron-specific enolase, tau, neurofilament M, neuron-specific nuclear protein, β-III-tubulin, and synaptophysin [202,203]. Pioneering studies also demonstrated the differentiation of BMMSCs into neural cell types *in vivo*. The transplantation of MSCs into mouse ventricles or striatum resulted in their expression of astrocytic traits [199,204]. It was shown for the first time that after the injection of mouse BMMSCs into the lateral ventricle of neonatal mice, MSCs migrated throughout the forebrain and cerebellum and differentiated into astrocytes in the striatum, the hippocampus and the reticular formation of the brain stem [199]. In addition, MSCs were found within neuron-rich regions and within the cerebellum [199]. The differentiation of MSCs into neurons was further confirmed in a study of the differentiation of mouse BMMSCs into phenotypic neural cells in ischemic animals [205]. Similarly, human BMMSCs implanted into ischemic rats, increased neurogenesis in these rats [206]. In addition, it was shown that human BMMSCs can differentiate into neural cells following their implantation in the brain of ischemic rats [207]. Moreover, the differentiation of MSCs into neurons was also demonstrated in the embryonic rat brain [208], and the stereotactic implantation of mouse MSCs into the brain of rats demonstrated the differentiation of implanted MSCs into mature neurons [209]. Recently, the transplantation of human placental MSCs into the striatum in a rat model of Parkinson’s disease also confirmed the ability of MSCs to differentiate into neurons [210]. Collectively, these studies support the ability of MSCs to differentiate into neurons.

**Figure 3:**
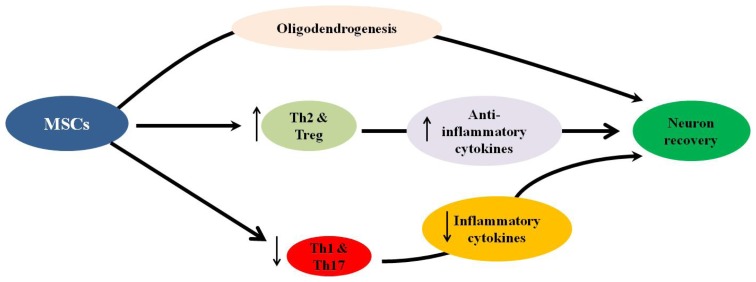
Mesenchymal stem cells can induce neuron recovery in multiple sclerosis via a mechanism that stimulates oligodendrogenesis and decreases the numbers of Th1 and Th17 cells and their secretion of inflammatory cytokines while increasing the numbers of Th2 and Treg cells and their secretion of anti-inflammatory cytokines.

The ability of BMMSCs to modulate the immunopathogenic process, leading to neuroprotection in EAE, was also demonstrated in several studies. It was shown that BMMSCs can improve neuronal recovery in EAE, possibly by stimulating oligodendrogenesis and reducing the inflammatory infiltrates, demyelination and axonal loss in the CNS of EAE mice by inhibiting autoreactive T cell responses [20,28,211,212]. In addition, the accumulation of BMMSCs in the tissues of the CNS and lymphoid organs of EAE mice reduced the severity of the disease by modulating the functions of T cells, as demonstrated by the following: (1) decreased inflammatory cytokine (INF-γ and IL-17) secretion by Th1 and Th17 cells; (2) increased numbers of Th2 cells and regulatory T cells and their secretion of anti-inflammatory cytokines; (3) decreased numbers of Th1 and Th17 T cells; and (4) increased numbers of oligodendrocytes in the CNS tissues of EAE mice (Figure 3) [17,21,26,27,213].

The neuroprotective effect of MSCs in EAE mice was further confirmed. It was shown that the administration of MSCs to EAE mice suppressed the clinicopathological manifestations of EAE and prevented axonal damage [23]. In this study, intraventricularly injected BMMSCs were detected in the inflamed CNS tissues of the EAE mice, and these MSCs exhibited features of neuronal lineages [23]. In addition, the intravenously injected MSCs migrated to the lymph nodes, which were associated with immunomodulatory effects, as demonstrated by the decrease in the immune cell infiltrate in the CNS [23]. Other studies confirmed the neuroprotective property of MSCs. The treatment of EAE mice with human endometrial MSCs significantly reduced EAE manifestations as a result of a reduction in Th1 and Th17 cell infiltrates in the CNS tissues of EAE mice [214]. This reduction in the target organ was probably a result of MSC-induced regulatory mechanisms in the periphery, as demonstrated by the up-regulation of IL-10, IL-27, immune suppressive enzyme, indoleamine-2,3-dioxygenase (IDO), and Foxp3 expression, thus indicating a higher percentage of putative Tregs [214]. In addition, BMMSCs that were transplanted intracerebroventricularly into EAE mice delayed the onset of symptoms and increased animal survival via mechanisms that possibly involved both immunomodulation and neuroprotection [215].

Recently, it was shown that with the intravenous administration of adipose-derived MSCs to EAE mice before disease onset, MSCs homed into lymphoid organs and migrated inside the CNS. These MSCs reduced the severity of EAE by immune modulation and decreased spinal cord inflammation and demyelination [18]. In addition, the administration of these adipose-derived MSCs to animals with chronic EAE ameliorated the disease course and reduced both demyelination and axonal loss while inducing Th2-type cytokine production [18]. Moreover, the infiltration of these MSCs within demyelinated areas was accompanied by increased numbers of endogenous oligodendrocyte progenitors [18]. The potential neuroprotective role of MSCs in EAE mice was also further confirmed. It was shown that human BMMSCs can migrate into the spinal cord in mice with EAE and that this significantly reduced the clinical disease severity [216]. The injected MSCs accumulated in the demyelinated areas, and these cells expressed neural markers [216]. In addition, the number of spinal cord white matter lesions and areas of white matter demyelination were reduced and were associated with decreased inflammatory infiltration after treatment with MSCs [216]. A recent study further confirmed the modulatory effects of MSCs on the immunopathogenic process in EAE. The transplantation of human placental MSCs into EAE mice yielded a decrease in disease severity in the transplanted animals via a mechanism that depends on the anti-inflammatory protein TNF-α-stimulated gene/protein 6 (TSG-6) [217].

These results demonstrate that MSCs have the therapeutic potential of suppressing the autoimmune response in early phases of disease and of inducing local neuroregeneration by endogenous progenitors in animals with established disease. In addition, MSCs were also shown to ameliorate the disease severity in EAE mice by the secretion of PGE2, which is dependent upon IDO for its immunosuppressive function [25]. The role of soluble factors in modulating the pathogenesis of EAE was confirmed in a recent study demonstrating that conditioned medium from human MSCs can reduce the functional deficits in EAE mice and can also promote the development of oligodendrocytes and neurons [218]. In agreement with this finding, it was found that human BMMSCs produce soluble factors that are important for mediating axon outgrowth and recovery in rats with injured spinal cords [219]. In addition, it was shown that BMMSCs can induce oligodendrocyte differentiation via factors produced by MSCs [220]. Moreover, when neural progenitor cells (NPCs), which were pre-differentiated *in vitro* by MSC-derived soluble factors, were transplanted *in situ* together with MSCs into hippocampal slice cultures, the grafted NPCs survived, and the majority of them differentiated into oligodendrocytes and neural progenitors [221]. Therefore, there is a general agreement regarding the effects of MSCs on improving the clinical manifestations of EAE in mice. However, the immunomodulatory properties of MSCs are not the only mechanisms that could explain their therapeutic plasticity. MSCs express a broad spectrum of regulatory proteins that may mediate their therapeutic functions. In addition, the ability of MSCs to respond to injuries depends on their microenvironment, regardless of whether they have a low engraftment rate *in vivo* [222]. MSCs produce cytokines and a variety of soluble factors that regulate several biological activities [191]. This suggests that MSCs can promote the survival of other cells and thus play a major role in the maintenance of tissue homeostasis [223].

### 4.2. Neuroprotective Properties of MSCs

The ability of MSCs to home to injured tissues and to transdifferentiate into multiple cell types *in vivo* was disputed by recent observations demonstrating that only small numbers of the injected MSCs can engraft into tissues and that the supernatant of MSC culture is sufficient to block hepatic failure [224,225]. In addition, several *in vitro* studies demonstrated that MSCs exert significant biological effects on target cells without the need for cell contact. Therefore, these and other *in vitro* and *in vivo* studies generated the hypothesis that the therapeutic effects of MSCs depend significantly on soluble factors secreted by MSCs and that these factors may mediate the MSC induction of tissue repair.

A number of *in vitro* studies showed that MSCs have the potential to rescue neurons from apoptosis [226–228]. Thus, this anti-apoptotic function of MSCs, together with their immunomodulatory properties, explain the reduction in axonal loss observed in EAE mice treated with MSCs [20,23,212]. In addition, MSCs secrete neurotrophic molecules, which would further explain how MSCs can induce remyelination in EAE mice [18,211,229].

The neuroprotective property of MSCs in EAE as discussed above was supported by several studies utilizing experimental models of stroke and spinal cord injury. For example, after the direct injection of BMMSCs into demyelinated spinal cords, they promoted remyelination [230]. In another study involving the transplantation of MSCs into the subarachnoid space of the lumbar spine, the MSCs infiltrated into the spinal cord parenchyma and then differentiated into immature neurons or glial cells. This was followed by complete transection and motor improvement [231]. In addition, BMMSCs were shown to improve the survival of motor neurons following their transplantation into the lumbar spinal cord in an animal model of human amyotrophic lateral sclerosis [232].

In a model of stroke, the intravenous administration of MSCs increased the expression of basic fibroblast growth factor, reduced apoptosis, promoted endogenous neurogenesis, and improved functional recovery [233]. MSCs can directly promote the plasticity of damaged neurons or stimulate glial cells to secrete neurotrophins (e.g., brain-derived neurotrophic factor (bDNF) and nerve growth factor (NGF)), reduce apoptosis in the penumbral zone of the lesion and support the proliferation of the endogenous cells in the subventricular zone [234]. In addition, the implantation of BMMSCs in the hippocampus of immunodeficient mice stimulated the proliferation, migration and differentiation of the endogenous neural stem cells, which survived as differentiated neural cells via their secretion of various trophic factors, including NGF, vascular endothelial growth factor (VEGF), ciliary neurotrophic factor (CNTF), basic fibroblast growth factor (FGF-2) and BMI-1 [235].

This indirect effect of MSCs on the improvement of neurogenesis was further supported in a mouse model of global ischemia, where MSCs improved neurological function and prevented neuronal cell death in the hippocampus via a mechanism that was mediated by the local microglia, which expressed high levels of neuroprotective factors, such as insulin-like growth factor 1 (IGF-1) [236]. This neuroprotective effect of MSCs on microglia was further confirmed. It was shown that MSCs can inhibit the production of inflammatory factors, including nitric oxide (NO), tumor necrosis factor, IL1-β and reactive oxygen species (ROS), by activated microglia, thus preventing neuronal damage via the production of neurotrophic factors, which are likely to be involved in neuroprotection [237]. In agreement with these results, it was reported that the cell death of dopaminergic neurons induced by activated microglia can be inhibited by MSCs [220,238]. In addition to the neuroprotective effects of MSCs on activated microglia, MSCs can significantly inhibit the up-regulation of molecules involved in oxyradical detoxification occurring in EAE [239]. Similarly, MSCs can resolve the increase of oxidative stress-associated proteins in neurons exposed to H_2_O_2_ [239]. This result suggests that MSCs can inhibit the neuroinflammatory process that is mediated by proinflammatory and oxidative stress molecules secreted by macrophages and microglia. However, microglia are not the only neural cell type targeted by MSCs; it was shown that MSCs can also inhibit the differentiation of neural precursor cells (NPCs) into astrocytes *in vitro* [220,229].

MSC transplantation represents an attractive therapeutic approach for MS treatment. However, many questions are essential to be answered before the use of MSCs in MS patients to determine their therapeutic potential. For example, it is essential to know the effect of ageing on MSC biology, since several studies indicate that ageing can affect the proliferation and differentiation capacities of MSCs. It has been shown that MSCs undergo replicative senescence, loss of differentiation capacity and ultimate growth arrest with increasing time in culture [240–250]. However, another study showed that aged MSCs have a higher proliferation rate than young MSCs [251] while another study did not find differences in the proliferation potential between aged and young MSCs [247]. These controversial results were attributed to species, gender and donor age of animals used in these studies and also to differences in the conditions of cell culture. However, these studies suggest that aging may change but not block the proliferation of MSCs. Regarding the differentiation potential of MSCs, the majority of studies reported an age-related reduction in the osteogenic potential [244,246,249–251]. In addition, this decrease in osteogenic capacity was associated with increased ability of aged MSCs to differentiate into adipocytes [244]. Since, these studies demonstrated that aging has an effect on the biological behaviours of MSCs following their long expansion time in culture; therefore this would not prevent their use in cell based therapies. More studies are necessary in order to determine the effects of aging on the immune- suppressive and neuroprotection functions of MSCs *in vitro* and *in vivo* before the use of MSCs in MS patients. It would be essential to compare the capacity of young *versus* aged MSCs in providing neuroprotection and neuroregeneration effects in EAE mice.

## 5. Conclusions

Evidence is increasing in support of the use of MSCs in treating neurological diseases, such as MS, via modulating the immunopathogenic process and promoting the repair of damaged neurons. In addition, the current data available in the literature suggest that the neurotherapeutic effect of MSCs is possibly mediated via a direct contact between MSCs and damaged neuronal cells or via soluble factors that are secreted by MSCs. MSCs secrete anti-inflammatory, anti-apoptotic and neurotrophic factors. These soluble factors can induce protective phenotypic features in other cells, such as microglia, and can also induce the remyelination process, thus maintaining the neuroprotective effects observed in experimental animal models of MS and other neurological disorder models. Therefore, these immunomodulatory and protective properties of MSCs may provide the basis to translate the neurotherapeutic effects of MSCs seen in animal models into humans in well-designed and carefully controlled clinical studies.
